# Perception of Shadows in Children with Autism Spectrum Disorders

**DOI:** 10.1371/journal.pone.0010582

**Published:** 2010-05-11

**Authors:** Cristina Becchio, Morena Mari, Umberto Castiello

**Affiliations:** 1 Centro di Scienza Cognitiva, Dipartimento di Psicologia, Università di Torino, Torino, Italy; 2 Dipartimento di Psicologia Generale, Università di Padova, Padova, Italy; University College London, United Kingdom

## Abstract

**Background:**

Cast shadows in visual scenes can have profound effects on visual perception. Much as they are informative, they also constitute noise as they are salient features of the visual scene potentially interfering with the processing of other features. Here we asked i) whether individuals with autism can exploit the information conveyed by cast shadows; ii) whether they are especially sensitive to noise aspects of shadows.

**Methodology/Principal Findings:**

Twenty high-functioning children with autism and twenty typically developing children were asked to recognize familiar objects while the presence, position, and shape of the cast shadow were systematically manipulated. Analysis of vocal reaction time revealed that whereas typically developing children used information from cast shadows to improve object recognition, in autistic children the presence of cast shadows—either congruent or incongruent—interfered with object recognition. Critically, vocal reaction times were faster when the object was presented without a cast shadow.

**Conclusions/Significance:**

We conclude that shadow-processing mechanisms are abnormal in autism. As a result, processing shadows becomes costly and cast shadows interfere rather than help object recognition.

## Introduction

Produced by the blockage of light from a light source by objects, cast shadows can provide valuable information about the presence and number, as well as the relative position of objects in the visual scene [1]. Furthermore, as they are images of the objects that cast them, they might be helpful in retrieving the 3D structure of objects [2] and recognizing objects [3]. Critically, in order to fulfil this function, cast shadows must be labelled by the visual system as shadows, i.e. as patterns of light, as opposed to permanent, independent features (shadow labelling problem). Furthermore, they must be linked with the objects that cast them (shadow correspondence problem) [4]. If these problems are not solved, observers will be confused by spurious dark patches in the image and will be unable to exploit information from cast shadows [5]. Different mechanisms have been proposed to explain how typical observers efficiently and rapidly process and identify regions as shadows [5]–[7]. The current investigation assessed how cast shadows are encoded by individuals with autism spectrum disorders.

Autism spectrum disorders are developmental disorders which are thought primarily to affect social functioning. However, there is now a growing body of evidence that attests to unusual sensory processing as least concomitant and, possibly the cause of some of the behavioural signs and symptoms associated with autism [8]. Literature on visual perception in autism has, for example, convincingly demonstrated superior performance in tasks requiring recognition of details [9], [10], ability to find hidden figures [11], [12], and visual search in feature and conjunctive search tasks [13]. By contrast, perception of dynamic and complex stimuli has been shown to be defective [14]. Hypotheses explaining such perceptual abnormalities include superior processing of low-level static information [15], [16], limited integration of low-level information in higher-order operations [17], and increased internal noise, potentially amplifying local differences and masking global differences [8].

Whereas there is some evidence that abnormalities in visual processing contribute to face processing difficulty in autism [18], it remains unclear whether anomalies in visual processing extend to other classes of visual objects. Processing of non-social objects is rather understudied in autism and to the extent that it has been studied, it is usually in the context of using non-face inputs as control stimuli for faces. Some studies claim that object processing abilities are spared in autism [19], [20]. Others suggest that individuals with autism might have problems with some types of object judgment [21], [22]. For example, observers with autism have been shown to have more difficulty with fine object discrimination compared to controls [21].

Although shadows are a fundamental feature of natural visual scenes, so far no study has examined whether object processing in autism is sensitive to their presence. Much as they are informative, shadows also constitute noise, as they are salient features of the visual scene potentially interfering with the processing of other features [5], [23]. Lighting under a particular set of conditions can produce shadows that may either help or hinder object recognition. Here, by comparing how autistic and typically developing children respond to the manipulation of the correspondence between objects and cast shadows, we aimed to answer two separate and yet strictly related questions: i) is recognition performance in autism sensitive to the presence of cast shadows? ii) are observers with autism especially sensitive to noise aspects of shadows?

## Methods

### Participants

Twenty high-functioning autistic children (ten males and ten females, 10–13 year old, mean 12.4 years) and 20 typically developing children (ten males and ten females, 10–13 year old, mean 12.2 years) with no reported neurological or academic problems participated in the study. All children were right-handed, reported normal or corrected-to-normal vision, no-hearing impairments, and were naive as to the purpose of the experiment. None was on medication or exhibited praxis problems as assessed by an occupational therapist. The children with autism were diagnosed according to the Diagnostic and Statistical Manual of Mental Disorders-IV (DSM-IV) criteria for autism. IQ was measured with the Wechsler Intelligence Scale for Children (WISC-R). The Childhood Autism Rating Scale [24] had been administered at the ages of 4–8 years by an experienced clinical psychologist. Further tools for diagnosis were the Autism Diagnostic Interview – Revised (ADI – R) [25] and the Autism Diagnostic Observation Schedule (ADOS) [26]. Only participants who met diagnostic criteria on the ADI-R and ADOS, as well as clinical judgment criteria were invited to participate. Participants with autism had no diagnoses of genetic syndromes or definable postnatal aetiologies for their developmental difficulties (e.g., head injury, tumour). At the time of the experiment, all of the children with autism were attending special education classes for autism. Participants were recruited from the community or from a database of families who had taken part in previous studies. Typically developing control participants had no history or evidence of autism on the ADI-R or ADOS, behavioural or psychiatric disorder as assessed by parent rating on the Child Behavior Checklist [27], no learning disabilities, and no history of head trauma. There were also no concerns about autism spectrum disorders in their first- or second-degree relatives. Participants with autism and control participants were matched by group on chronological age, full scale IQ, socioeconomic status [28], gender and handedness (see Table 1 for the participants' descriptive characteristics). This research was approved by the ethical committee of the Università di Padova and was conducted according to the Declaration of Helsinki. Before testing, the participants' parents gave their written informed consent. The participants also gave their written consent.

**Table 1:** Descriptive characteristics for the autism and the typically developing (TD) groups.

	Autism	TD	*F* or *χ2*	*p*
*n*	20	20	-	-
Age	12.4 (2.0)	12.2 (1.66)	.05	.64
Full Scale IQ	102.4 (12.41)	108.5 (10.61)	1.32	.15
Socioeconomic Status	53.21 (8.21)	54.15 (7.56)	.32	.41
Handedness (R:L)	20:0	20:0	.24	.33
Gender (M:F)	10:10	10:10	.21	.42
CARS	34.1 (4.92)	-	-	-

Means and standard deviations (in parentheses) are shown along with corresponding *F* or *χ2* values and p values for between group comparisons. Notes: CARS, Childhood Autism Rating Scale.

### Materials

**Figure 1:**
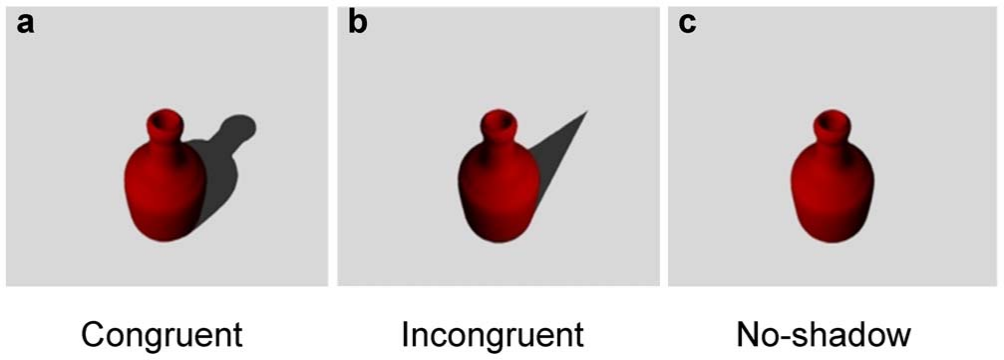
Examples of images used to depict the various object–shadow combinations. The shape of the shadows could be either congruent (A) or incongruent (B) with the shape of the objects. Shadows were presented to both the right and the left of the objects. Panel C depicts an object (a bottle) without a shadow.

Stimuli consisted in familiar objects chosen for their strong geometrical properties (for an example see Fig. 1). The objects depicted were: apple, banana, bottle, calculator, can, cross, cylinder, eraser, fork, glass, glove, jug, knife, mandarin orange, mug, pen, pyramid, sphere, tennis racket, and vase.

They were synthesized using the 3D rendering package POV-Ray (Persistence of Vision Raytracer). When generating the digital images, the objects were positioned at the origin of an imaginary set of (x, y, z) axes, with y pointing orthogonally out of the image (i.e. towards the subjects), and x and z the horizontal and vertical axes respectively. The camera was positioned along the y axis so that it looked down upon the objects at an angle of 45 degrees. The objects were illuminated with ambient and point light sources either from the right or from the left in order to avoid the effects of up/down illumination changes on perceived shape. The right and left light sources were located at +/− 34 degrees along the x-y plane respectively, again pointing down upon the objects at an angle of 45 degrees. The reflectance model used an ambient reflectance of 0.2. The final images used as stimuli were created by digitally combining shadows and object images. Shadow images were generated by moving the objects towards the light sources, out of the camera's field of view. The objects were then scaled so that these (generated) shadows were in proportion to the original objects. All subjects viewed the objects binocularly from a distance of approximately 70 cm. The area subtended by the objects, including the shadows, was 7.8×7.8 degrees of visual angle.

### Experimental conditions and procedure

The following experimental conditions were tested: i) congruent condition, in which an object was presented with its naturally cast shadow; ii) incongruent condition, in which an object was presented with a cast shadow originating from another object; iii) no-shadow condition, in which an object was presented without a cast shadow. Please note that in the no-shadow condition the objects were still presented with lighting coming either from the right or from the left as for the conditions in which the shadow was present. Participants initiated a trial by depressing a start button. An object (with or without a shadow, depending on the condition) would appear in the centre of the screen and they were required to report the identity of the presented object as quickly as possible. The vocal response time (VRT) was taken from the moment the stimulus first appeared to the instant in which the subject emitted an audible vocal response, detected by means of a voice-key. The end of the trial was taken as either the time of the vocal response or 2000 ms after the stimulus presentation if no response was made. The subsequent trial was presented after an interval of 2000 ms. Each participant first completed 20 practice trials, which were followed by 4 blocks of 60 trials. Each block consisted of 20 trials for each condition (10 trials in which the objects were illuminated from the right; 10 trials in which the objects were illuminated from the left) presented in a randomized order. The duration of each block was no longer than 20 minutes and all blocks were separated by a rest period of 5–10 minutes. Trials in which errors of anticipation (i.e. reaction times of less than 150 ms) occurred, no response was made, or the responses were made after 2000 ms had elapsed were automatically re-set to the end of the block to be re-presented in a random order. Catch trials, in which no object appeared, were also included in order to prevent expectancy and/or practice effects.

Before the experiment started all subjects attended a preliminary session in which they were presented with and asked to recognize the objects from which the stimuli were derived. All subjects were able to recognize and verbally report the name of the presented objects. Because of the vocal response modality, it was not possible to code each trial for accuracy during the experiment. Accuracy scores are therefore not reported.

### Data analysis

A preliminary analysis of variance (ANOVA) was conducted to verify possible differences for shadows presented to the right or to the left of the object. Here the main factor was shadow position (right, left). No differences in vocal reaction time were found depending on shadow position (p>0.05). This allowed us to collapse data for right and left shadow and to perform an ANOVA with group (autistic, typically developing) as a between-subjects factor and experimental condition (congruent, incongruent, control) as a within-subjects factor. Bonferroni corrections (alpha level, p<0.05) were applied for the contrasts of interest.

## Results

**Figure 2:**
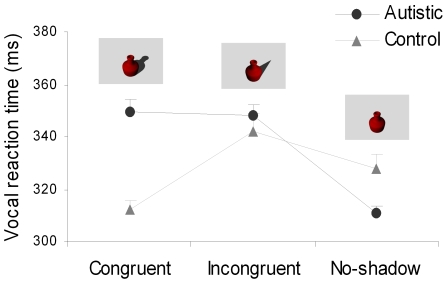
Graphical representation for the interaction between group (autistic, typically developing) and experimental condition (congruent, incongruent, no-shadow). The error bars correspond to the standard errors of the means.

The group by experimental condition interaction was significant [F(2,76) = 31.14, p<0.0001; Mean square: 7558.3]. There was a significant main effect of condition [F(2,76) = 27.87, p<0.0001; Mean square: 6765]; at the same time, no significant main effect of group was evident [F(2,76) = 2.74, p = .106; Mean square: 2323.2], determining the crossover shape observed for the interaction (see Figure 2). Post-hoc contrasts revealed that for typically developing children VRT was significantly faster for the congruent than for both the incongruent (p<0.0001) and the no-shadow (p<0.005) conditions (see Figure 2). Furthermore, VRT was longer for the incongruent than for the no-shadow condition (p<0.0001). For children with autism, VRT for the no-shadow condition was faster than for either the congruent or the incongruent conditions (p_s_<0.0001). VRT was similar for the congruent and the incongruent conditions (p>0.05).

## Discussion

Much as they are informative, cast shadows also constitute noise as they are salient features of the visual scene (because of high luminance contrast at their boundary) and it takes very little to make them look like independent surface features (e.g., by drawing a line at their boundary which increases the luminance contrast) [5], [23]. Our study demonstrates how in autism noise aspects of cast shadows prevail over informativeness. Whereas typically developing children use information from cast shadows, in autistic children the presence of cast shadows – either congruent or incongruent – interferes with object recognition.

Typically developing children are slower to recognize objects in the presence of incongruent cast shadows, but also when presented with objects without cast shadows. In line with previous results [3], this finding supports the idea of an *implicit shadow mechanism* that can rapidly identify changes in illumination as shadows and allows information contained in cast shadow to be used for recognition purposes [6]. It has been proposed that this mechanism might operate at a coarse visual scale [5] and this would possibly explain why small discrepancies in shadows tend to be ignored, i.e. why typical observers are insensitive to small changes in the shape and angular orientation of shadows [7]. However, when the discrepancies become larger, the situation changes radically. Shadows are no longer processed implicitly as such and turn into highly detectable objects, potentially interfering with the processing of other visually available objects. We propose that performance of typically developing children might be explained in these terms, assuming that shadows are labelled by the visual system as shadows for the congruent, but not for the incongruent condition. This would explain why in typically developing children congruent shadows determine facilitation, whereas incongruent shadows determine interference with object processing, slowing down the vocal response.

The cost found for congruent cast shadows in autism suggests that the shadow processing mechanism might be abnormal in this population. Critically, children with autism did not appear generically distracted by changes in illumination: in fact, changes in illumination were also present for the no-shadow condition, in which the objects were presented with lighting coming either from the right or from the left as for the conditions in which cast shadows were present. If the performance of autistic children reflected a higher level of distraction by the constant changing of illumination, a generic increase in response time might be expected across conditions. The lack of a main effect of group strongly argues against this possibility. Furthermore, because the position of cast shadows changed randomly for the congruent as well as for the incongruent condition, the observed pattern of results is unlikely to reflect shifting of shadow position. If children with autism were distracted by the mere change in the position of shadows, a slowing down in VRT with respect to that of typically developing controls should have been observed for both congruent and incongruent shadows. In contrast, whereas VRT of autistic children was significantly slower compared to that of typically developing children for the congruent condition (p<.0001), no difference in performance was observed between autistic and typically developing controls for the incongruent condition (p>0.05).

These findings are indicative of specific impairment in the processing of congruent shadows.

In typical observers, objects and congruent cast shadows are linked to improve recognition and shadow processing becomes costly only when large discrepancies prevent dark areas from being recognized as shadows [3]. In children with autism, object recognition is faster when no cast shadow is present: both congruent and incongruent shadows interfere with object recognition.

A possible explanation lies in the saliency of cast shadows: cast shadows are salient features. For autistic observers they might become hyper-salient. In autism, enhanced sensitivity for specific stimuli has been demonstrated in different sensory modalities [29]. In vision, Bertone and colleagues [14] found that individuals with autism obtained significantly lower thresholds for a static contrast sensitivity task. Specifically, the ability of observers with autism was found to be superior for identifying simple, luminance-defined (or first-order) contrasts but inferior for complex, texture-defined (or second-order) contrasts. As cast shadows are changes in illumination, it might well be that lower thresholds for luminance contrasts increase the sensitivity of observers with autism to cast shadows, turning them into hyper-salient features. Congruent shadows might no longer be processed implicitly as shadows and linked to the objects that cast them, but treated as independent features, potentially competing with the processing of objects in the scene. This would explain why in autism the processing of congruent shadows results as costly as the processing of incongruent shadows.

Another possibility is that abnormal processing of shadows reflects reduced top-down modulation of visual attention. Models of visual attention incorporate two different sources for driving attention: simple features such as high contrast or motion that influence the allocation of attention in a bottom-up fashion and top-down information gained from the knowledge of the structure and meaning of the stimulus [30]. Critically, high-level structural knowledge has been shown to constrain and guide shadow perception in typical observers independently of guidance by low-level visual processes [31]. In autism, influence of structural knowledge on shadow perception might be reduced by problems in high-level mechanisms of attention [17], [32]. In this interpretation, observers with autism might be distracted by shadows – either incongruent or congruent - because, as a consequence of reduced top-down modulation, their attention tends to be grabbed by low-level visually salient features.

The idea that individuals with autism see the world differently - for example, easily perceiving elements that might remain hidden from the experience of typical observers - is perhaps the most intriguing of all the puzzles thrown up by autism [17]. The present results provide a notable demonstration of how the consequences of visual vagaries in autism may extend beyond vision. Whereas typically developing children used information from cast shadows to improve object recognition, in autism abnormal visual processing transforms shadows into ‘dark things’ that interfere rather than help object recognition. Future studies will have to understand the role that problems in low-level mechanisms of basic perception and high-level mechanisms of attentional modulation play in this process.
